# Supplementary material for: Female sex worker preferences for HIV pre-exposure prophylaxis delivery in Uganda: A discrete choice experiment
Source: PLOS Glob Public Health. 2025 Jun 24;5(6):e0003848. doi: 10.1371/journal.pgph.0003848 (PMC12186891; doi:10.1371/journal.pgph.0003848)
Supplement: S1 File — (PDF) [file pgph.0003848.s001.pdf]

TOOL TO ASSESS FEMALE SEX WORKERS (FSW) PREFERENCES FOR DIFFERENT COMPONENTS OF A PRE-EXPOSURE PROPHYLAXIS (PrEP) DELIVERY MODEL IN UGANDA

FSW INTERVIEW TOOL

1. Name of Research Assistant: ....

   ....

2. Health Facility: ....

   ....

3. District: ....
4. Region: ....
5. Date of Review: ....

FSW DEMOGRAPHICS

6. PrEP No: ....
7. Age (Completed Years): ....
8. Marriage Status *(Tick against the correct option)*

   Single .... Married .... Divorced/Separated ....

9. Other occupation: ....
10. Education Level: None .... Primary .... Secondary ....

    Diploma .... Degree .... *(Tick against the correct option)*

11. Area of Residence: Within Kampala .... Outside Kampala ....

FSW MEDICAL HISTORY

12. Date initiated on PrEP: ....
13. Duration on PrEP *(Tick against the correct option)*

    a) Less than 6 months ....

    b) Less than 1 year .... c) More than 1 year ....

14. Current way of getting PrEP *(Tick against the correct option)*

    a) Community .... b) Facility .... c) Both ....

15. Any underlying co-morbidities *(Tick against the correct option)*

    No .... Yes ....

16. If yes *(Tick against the correct option)*

    Diabetes .... Hypertension ....

    Others, Specify

    ....

    ....

    ....

    ....

17. Elicit individual FSW preferences (pairwise choices) and document in the spaces provided for each table for all the pairs.

Option 1
HOTSPOT

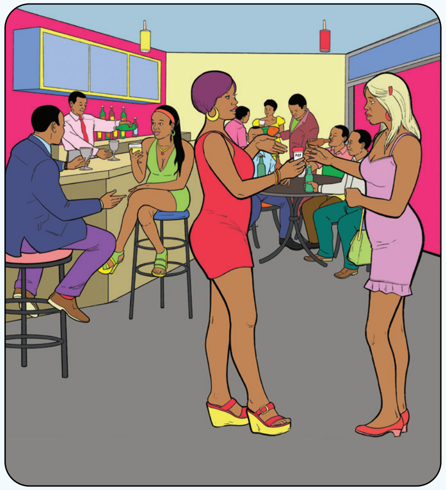

Sex worker receiving PrEP from a peer at a hotspot.

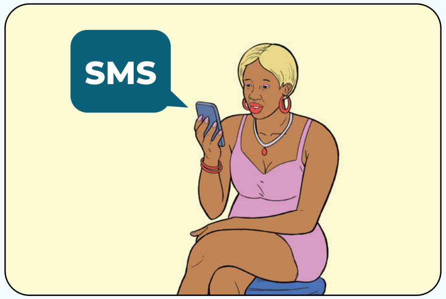

Sex worker receiving SMS reminders about taking PrEP

Option 2
HOME

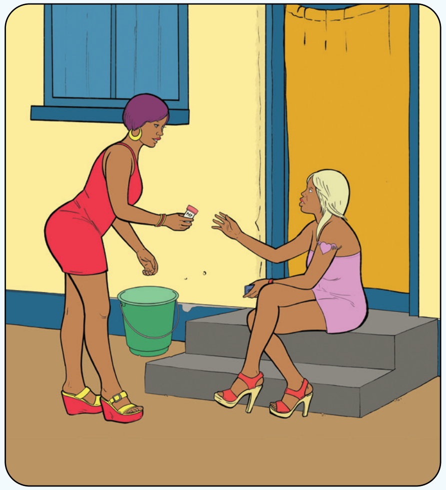

Peer delivering PrEP to a sex worker at her home.

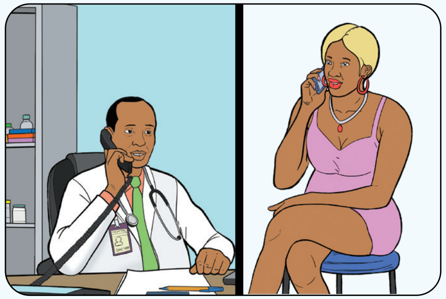

Sex worker receiving call reminders about taking PrEP.

Option 3
COMMUNITY

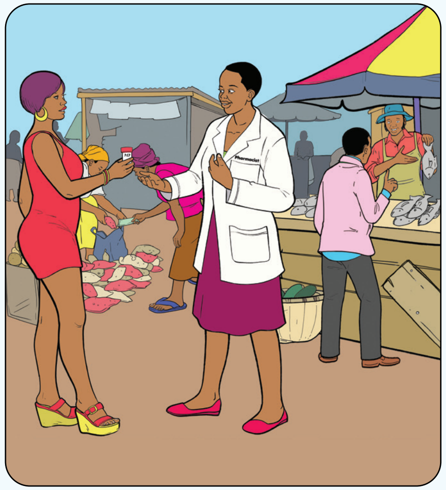

Peer receiving PrEP from a pharmacist on behalf of the sex worker in the community.

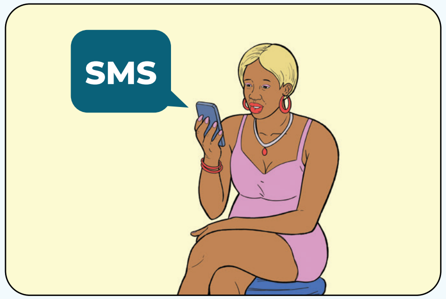

Sex worker receiving SMS reminders about taking PrEP.

Option 4
NEITHER

CARD A

Write in this box the option number for the preferred modality of extending PrEP to you.

Option 1

HOME

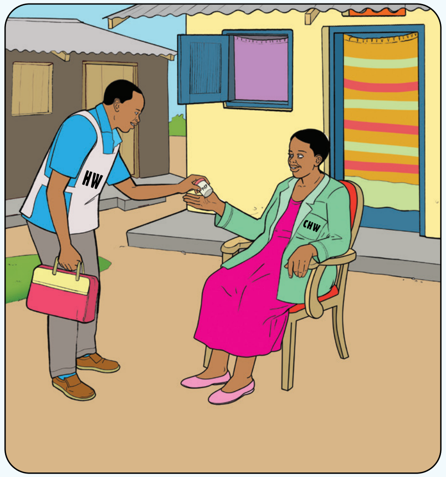

Community Health Worker (CHW) receiving PrEP from a Health Worker (HW) on behalf of the sex worker at home.

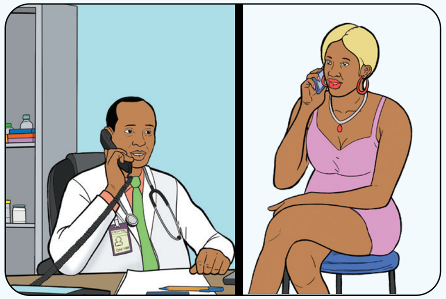

Sex worker receiving call reminders about taking PrEP.

Option 2

COMMUNITY

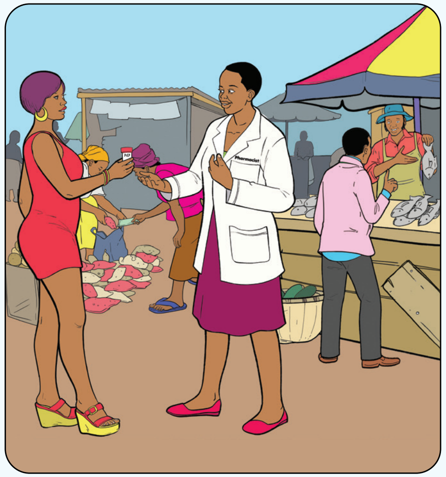

Peer receiving PrEP from a pharmacist on behalf of the sex worker in the community.

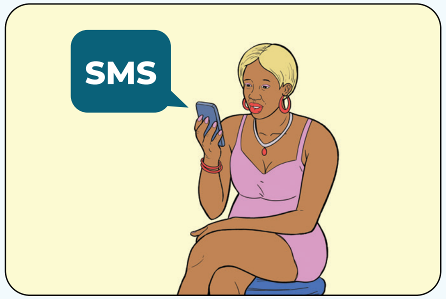

Sex worker receiving SMS reminders about taking PrEP.

Option 3

HEALTH FACILITY

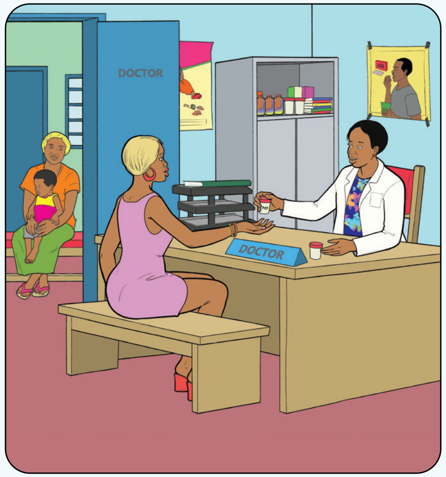

Sex worker receiving PrEP from a health facility in person.

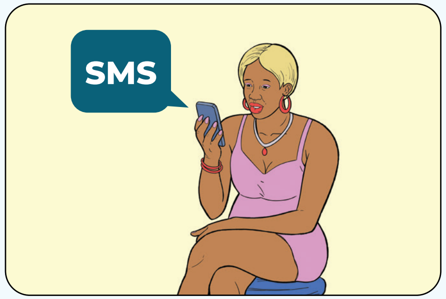

Sex worker receiving SMS reminders about taking PrEP.

Option 4

NEITHER

CARD B

☐ *Write in this box the option number for the preferred modality of extending PrEP to you.*

Option 1
HOTSPOT

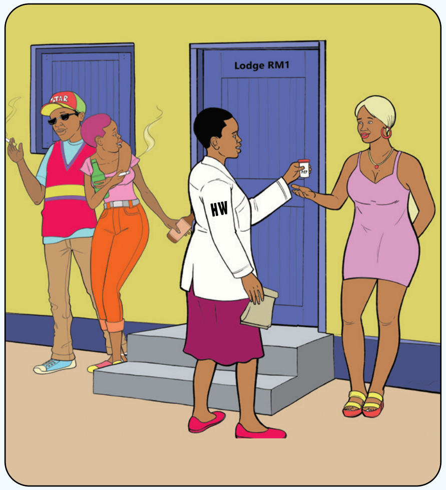

Healthworker delivering PrEP to a sex worker at a hotspot.

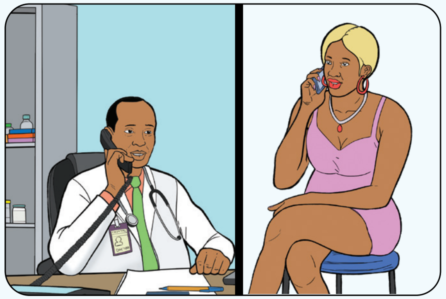

Sex worker receiving call reminders about taking PrEP.

Option 2
HEALTH FACILITY

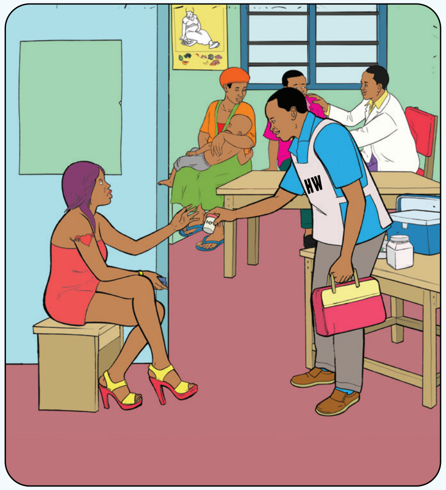

Peer receiving PrEP on behalf of the sex worker from health worker at the health facility.

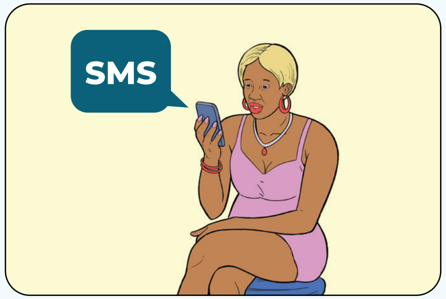

Sex worker receiving SMS reminders about taking PrEP.

Option 3
COMMUNITY

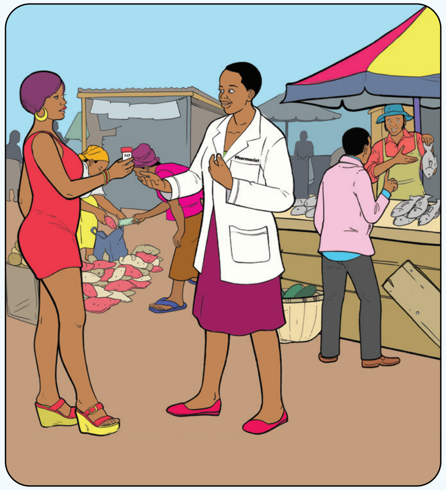

Peer receiving PrEP from a pharmacist on behalf of the sex worker in the community.

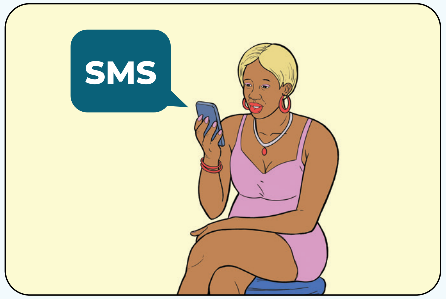

Sex worker receiving SMS reminders about taking PrEP.

Option 4
NEITHER

CARD C

Write in this box the option number for the preferred modality of extending PrEP to you.

Option

1

HOME

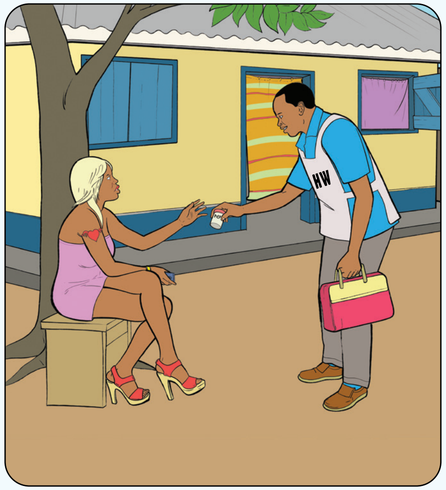

Sex worker receiving PrEP from a health worker in person at home.

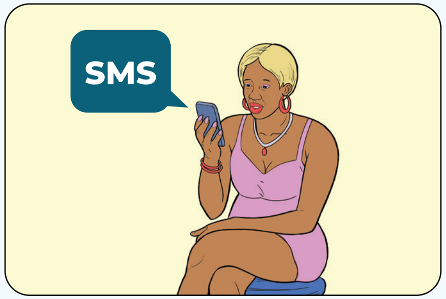

Sex worker receiving SMS reminders about taking PrEP.

Option

2

COMMUNITY

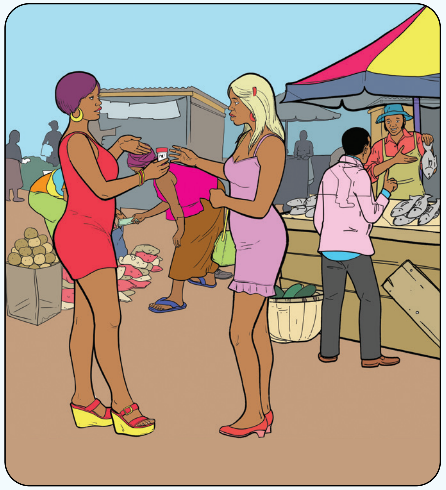

Peer delivering PrEP to a sex worker in the community.

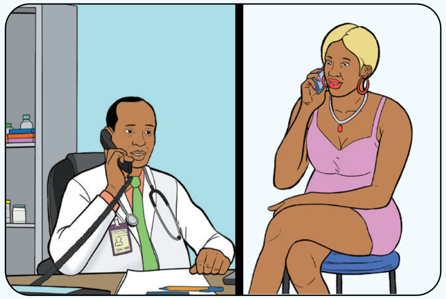

Sex worker receiving call reminders about taking PrEP.

Option

3

COMMUNITY

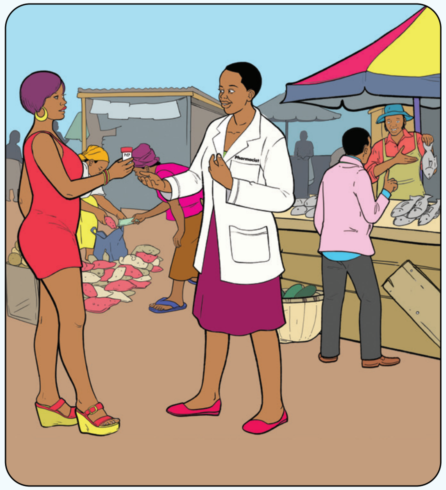

Peer receiving PrEP from a pharmacist on behalf of the sex worker in the community.

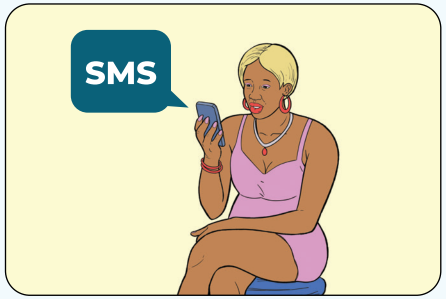

Sex worker receiving SMS reminders about taking PrEP.

Option

4

NEITHER

CARD D

☐ *Write in this box the option number for the preferred modality of extending PrEP to you.*

Option 1
HEALTH FACILITY

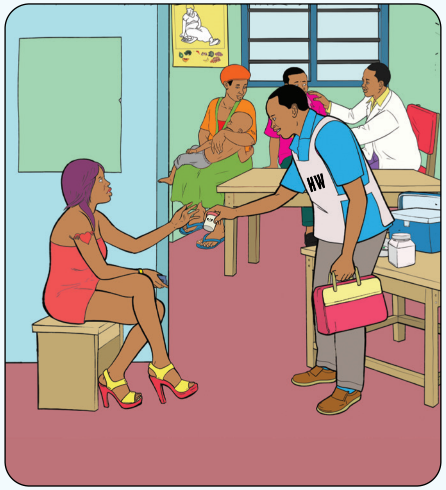

Peer receiving PrEP on behalf of the sex worker from health worker at the health facility.

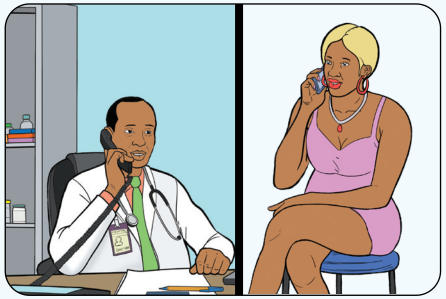

Sex worker receiving call reminders about taking PrEP.

Option 2
COMMUNITY

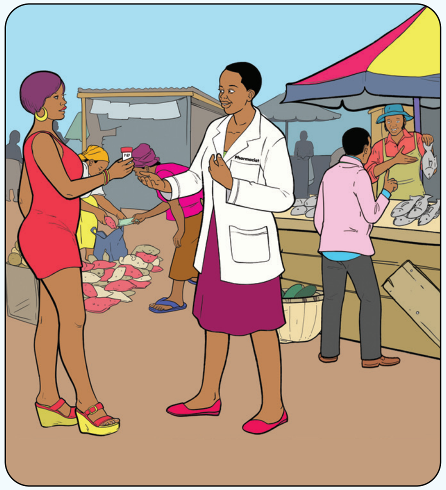

Peer receiving PrEP from a pharmacist on behalf of the sex worker in the community.

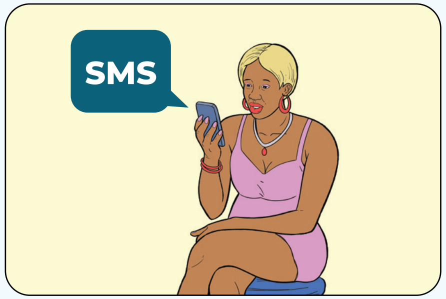

Sex worker receiving SMS reminders about taking PrEP.

Option 3
HOTSPOT

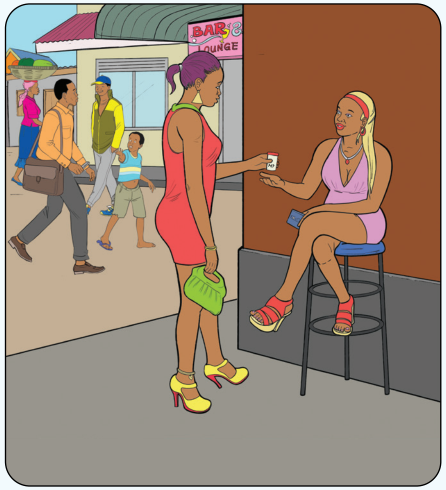

Sex worker receiving PrEP in person from a peer at a hotspot.

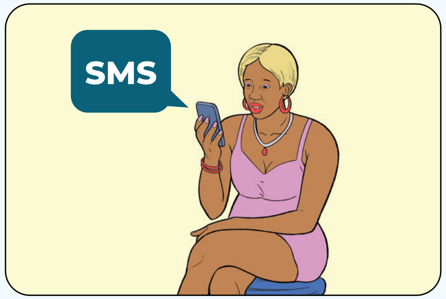

Sex worker receiving SMS reminders about taking PrEP.

Option 4
NEITHER

CARD E

☐ *Write in this box the option number for the preferred modality of extending PrEP to you.*

Option 1
HEALTH FACILITY

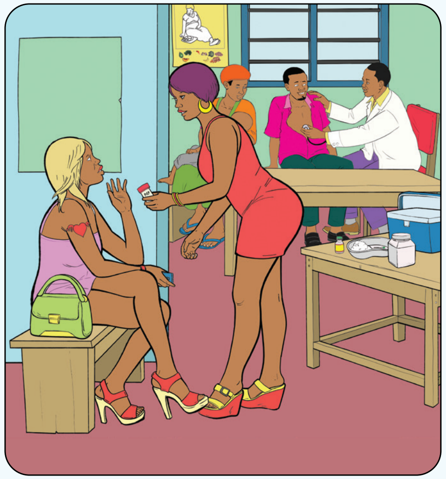

Sex worker receiving PrEP in person from a peer at a health facility.

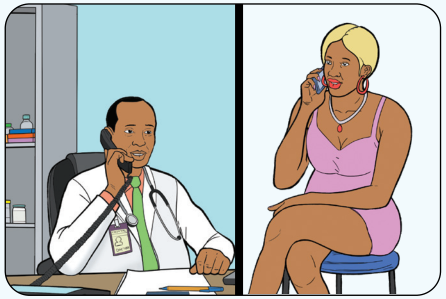

Sex worker receiving call reminders about taking PrEP.

Option 2
HOTSPOT

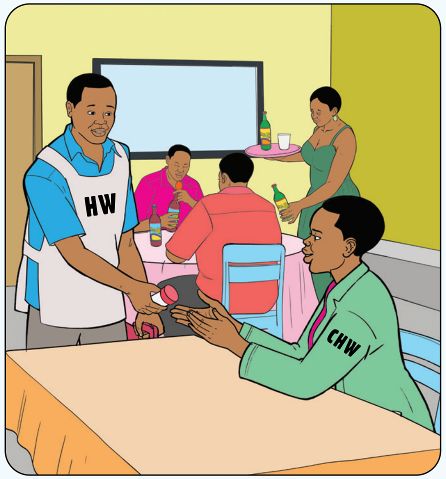

Community Health Worker (CHW) receiving PrEP from a Health Worker (HW) on behalf of the sex worker at a hotspot.

None

Option 3
COMMUNITY

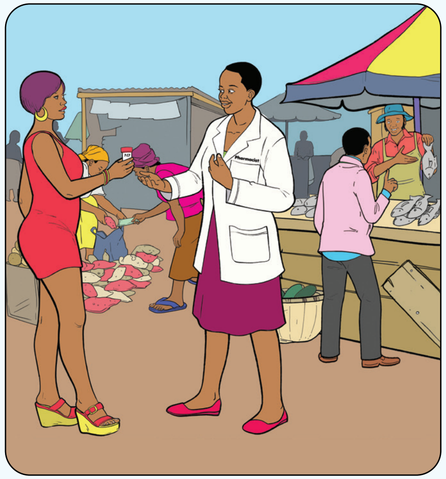

Peer receiving PrEP from a pharmacist on behalf of the sex worker in the community.

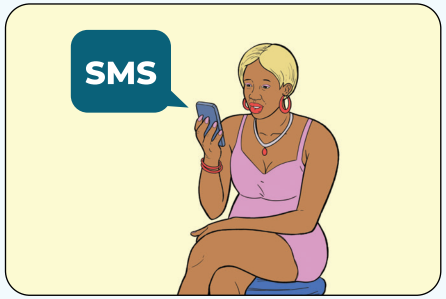

Sex worker receiving SMS reminders about getting PrEP

Option 4
NEITHER

CARD F

☐ *Write in this box the option number for the preferred modality of extending PrEP to you.*

Option

1

COMMUNITY

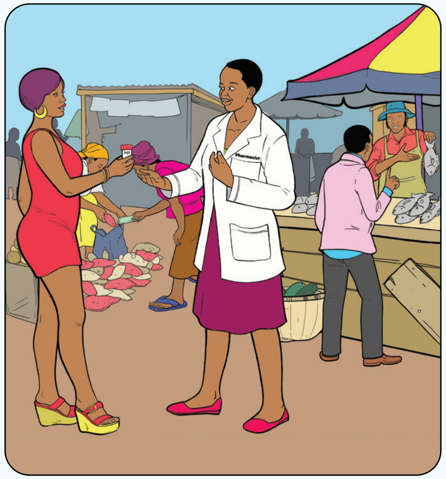

Peer receiving PrEP from a pharmacist on behalf of the sex worker in the community.

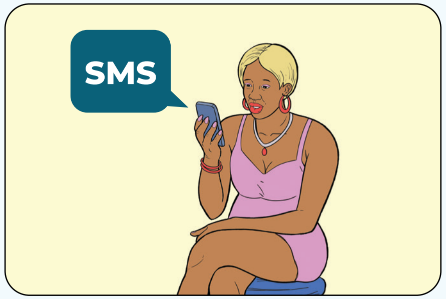

Sex worker receiving SMS reminders about taking PrEP.

Option

2

HOME

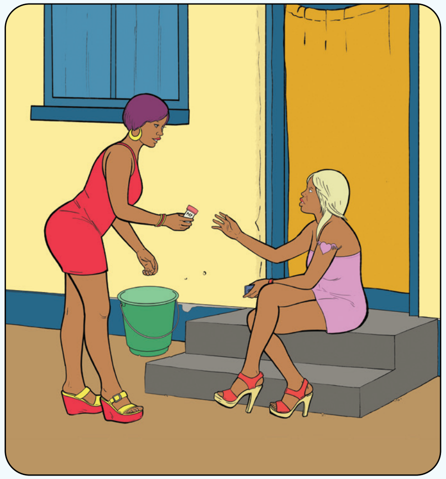

Peer delivering PrEP to a sex worker at her home.

None

Option

3

HOTSPOT

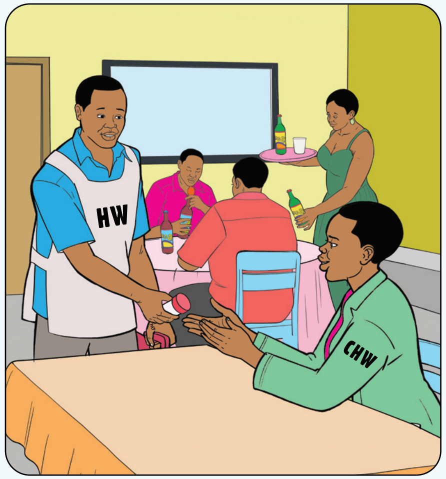

Community Health Worker (CHW) receiving PrEP from a Health Worker (HW) on behalf of the sex worker at a hotspot.

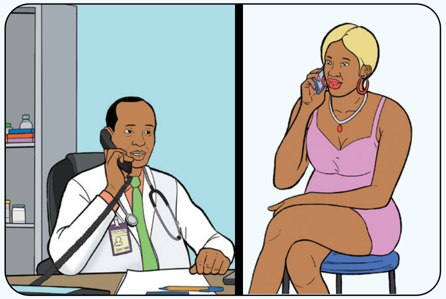

Sex worker receiving call reminders about taking PrEP.

Option

4

NEITHER

CARD G

Write in this box the option number for the preferred modality of extending PrEP to you.

Option 1

COMMUNITY

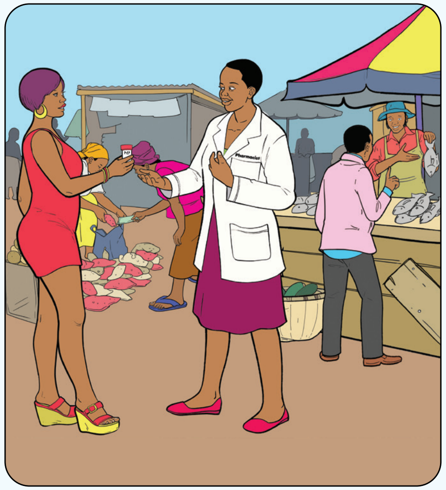

Peer receiving PrEP from a pharmacist on behalf of the sex worker in the community.

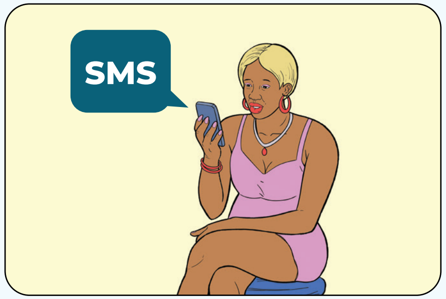

Sex worker receiving SMS reminders about taking PrEP.

Option 2

HOME

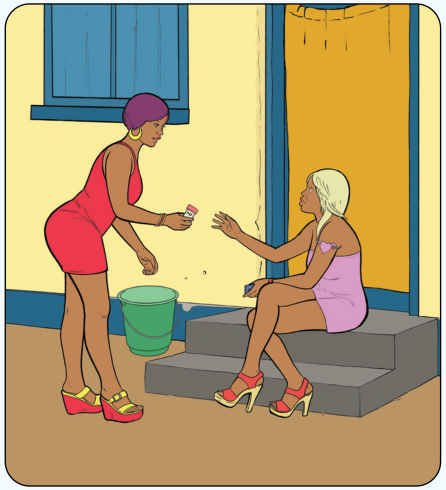

Peer delivering PrEP to a sex worker at her home.

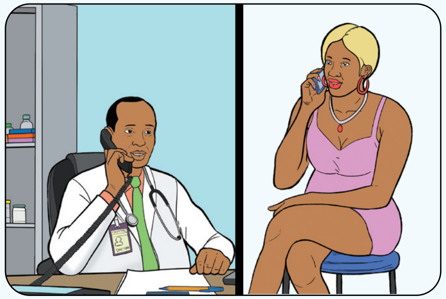

Sex worker receiving call reminders about taking PrEP.

Option 3

HOTSPOT

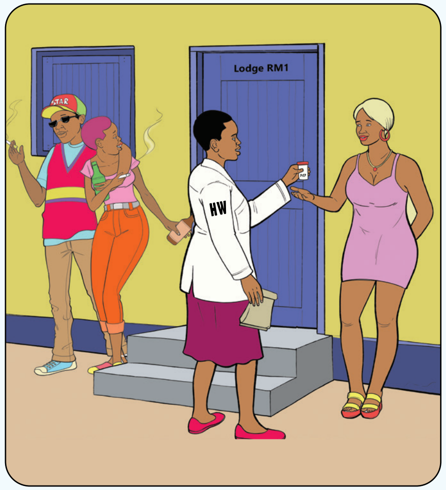

Healthworker delivering PrEP to a sex worker at a hotspot.

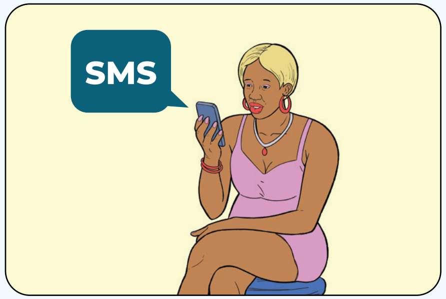

Sex worker receiving SMS reminders about taking PrEP.

Option 4

NEITHER

CARD H

☐ *Write in this box the option number for the preferred modality of extending PrEP to you.*
