# Supplementary material for: Female sex worker preferences for HIV pre-exposure prophylaxis delivery in Uganda: A discrete choice experiment
Source: PLOS Glob Public Health. 2025 Jun 24;5(6):e0003848. doi: 10.1371/journal.pgph.0003848 (PMC12186891; doi:10.1371/journal.pgph.0003848)
Supplement: Attachment — Submitted filename: Response to Reviewers 05.05.2025.docx [file pgph.0003848.s004.docx]

# Makerere University

P. O. Box 7072 Kampala, Uganda	Tel: 256 414 530022

E-mail: ceu@chs.mak.ac.ug	Fax: 256 414 532203

**CLINICAL EPIDEMIOLOGY UNIT SCHOOL OF MEDICINE**

02 May 2025

Nazmul Alam, MPH, DrPH
Academic Editor
PLOS Global Public Health

**Regarding: PGPH-D-24-02264 Female Sex Worker Preferences for HIV Pre-Exposure Prophylaxis Delivery in Uganda: A Discrete Choice Experiment**

We thank the editor and the reviewers for their constructive feedback, which has enhanced the quality of the paper. Please find our responses below in italics, with revised text in bold font:

**Journal requirements**

1. Please amend your detailed Financial Disclosure statement. This is published with the article. It must therefore be completed in full sentences and contain the exact wording you wish to be published.

**Please only choose the relevant sentences from below**

i. Please clarify all sources of funding (financial or material support) for your study. List the grants (with grant number) or organizations (with url) that supported your study, including funding received from your institution.

ii. State the initials, alongside each funding source, of each author to receive each grant.

iii. State what role the funders took in the study. If the funders had no role in your study, please state: “The funders had no role in study design, data collection and analysis, decision to publish, or preparation of the manuscript.”

iv. If any authors received a salary from any of your funders, please state which authors and which funders.

***Response:*** *Thank you for highlighting this. The Financial Disclosure statement has been amended, per your guidance, to include the details below (page 18, lines 407 - 415).*

**Funding**

**Fogarty International Center, National Institute of Alcohol Abuse and Alcoholism, National Institute of Mental Health, of the National Institutes of Health under Award Number D43 TW011304, and Makerere University Research and Innovations Fund (MakRIF) under award Number PhD round 4 supported the research reported in this publication. The funders had no role in study design, data collection and analysis, decision to publish, or preparation of the manuscript. The content is solely the authors' responsibility and does not necessarily represent the official views of the National Institutes of Health.**

2. Please make sure the funding information on the submission form matches your financial disclosure statement. Please indicate by return the full and correct funding information for your study and confirm the order in which funding contributions should appear. Please be sure to indicate whether the funders played any role in the study design, data collection and analysis, decision to publish, or preparation of the manuscript.

**Response**: *Thank you for bringing this to our attention. Per your guidance, the Financial Disclosure statement has been amended to include the details below (page 18, lines 407 - 415).*

**Funding**

**Fogarty International Center, National Institute of Alcohol Abuse and Alcoholism, National Institute of Mental Health, of the National Institutes of Health under Award Number D43 TW011304, and Makerere University Research and Innovations Fund (MakRIF) under award Number PhD round 4 supported the research reported in this publication. The funders had no role in study design, data collection and analysis, decision to publish, or preparation of the manuscript. The content is solely the authors' responsibility and does not necessarily represent the official views of the National Institutes of Health.**

3. In the online submission form, you indicated that “The datasets used and analyzed during the current study are available from the corresponding author on reasonable request.”

All PLOS journals now require all data underlying the findings described in their manuscript to be freely available to other researchers, either

1. In a public repository,

2. Within the manuscript itself, or

3. Uploaded as supplementary information.

This policy applies to all data except where public deposition would breach compliance with the protocol approved by your research ethics board. If your data cannot be made publicly available for ethical or legal reasons (e.g., public availability would compromise patient privacy), please explain your reasons by return email and your exemption request will be escalated to the editor for approval. Your exemption request will be handled independently and will not hold up the peer review process, but will need to be resolved should your manuscript be accepted for publication. One of the Editorial team will then be in touch if there are any issues.

**Response**: *Thank you! This is well noted. We have uploaded the data as supplementary information.*

4.Some material included in your submission may be copyrighted. According to PLOS’s copyright policy, authors who use figures or other material (e.g., graphics, clipart, maps) from another author or copyright holder must demonstrate or obtain permission to publish this material under the Creative Commons Attribution 4.0 International (CC BY 4.0) License used by PLOS journals. Please closely review the details of PLOS’s copyright requirements here: PLOS Licenses and Copyright. If you need to request permissions from a copyright holder, you may use PLOS's Copyright Content Permission form.

Please respond directly to this email or email the journal office and provide any known details concerning your material's license terms and permissions required for reuse, even if you have not yet obtained copyright permissions or are unsure of your material's copyright compatibility.

Potential Copyright Issues:

Supplementary File 1.docx.pdf: Please confirm whether you drew the images / clip-art within the figure panels by hand. If you did not draw the images, please provide (a) a link to the source of the images or icons and their license / terms of use; or (b) written permission from the copyright holder to publish the images or icons under our CC-BY 4.0 license. Alternatively, you may replace the images with open source alternatives. See these open-source resources you may use to replace images / clip-art:

- [https://commons.wikimedia.org](https://commons.wikimedia.org/)

- <https://openclipart.org/>

**Response**: *Thank you for your comment. We confirm that the images in Supplementary File 1 are original illustrations. They were initially hand-drawn on paper, scanned in black and white, and later enhanced using Photoshop with the assistance of a graphics designer (Mr. Michael Kateregga, Tel: +256777099492, Email: mycolkm@gmail.com). The enhancements included adding color and refining the outlines for improved clarity. These images were specifically customized to this study by the corresponding author, who retains full rights to use them in any document and as many times as needed.*

*.*

**REVIEWER 1**

Well written and informative article. I have learned a lot from it.

**Response**: *We thank the reviewer for this favorable comment.*

Minor revision: One of the two major findings is the clear preference of FSWs for receiving PrEP from competent and supportive health workers. I was unable to get a sense from the article whether there are workforce challenges such as shortages or maldistribution in Uganda in particular the workforce that provides such care for FSWs. I agree with the authors emphasizing the need for supplementary support mechanisms such as implementing SMS reminders. However, optimization of the workforce in terms of training and retention is not discussed or put forth as a recommendation. From a health system perspective, this is an excellent opportunity to advocate for upscaling the workforce in order to implement more such programs throughout the country to serve more FSWs. I suggest adding this to the discussion and recommendations.

**Response**: *Thank you for this insightful recommendation. We have revised the conclusion and recommendation sections to include the importance of optimizing the workforce through targeted training and retention strategies. This section now reads as below (pages 16-17, lines 376 - 393).*

**Conclusions and recommendations**

**FSWs showed a clear preference for receiving PrEP directly from a health worker within a friendly and supportive healthcare setting. To enhance adherence and mitigate the risk of adverse health outcomes like HIV acquisition due to non-adherence, there is a significant need for supplementary support mechanisms. Implementing SMS reminders could improve adherence rates and ensure better health outcomes for FSWs using PrEP. These preferences should be considered when designing future approaches for delivering PrEP through the National PrEP Program. Optimization of health worker training and retention should be prioritized as a key component of health system improvements. This should include advocating for the recruitment of an adequate number of health workers to effectively implement and expand programs nationwide, ensuring broader access to services for FSWs, and enhancing the overall impact of such initiatives. As mentioned above,** **further evaluation of the feasibility and effectiveness of this PrEP delivery model is necessary, including in vulnerable populations, such as pregnant FSWs. A study is underway to determine the feasibility and acceptability of this preferred PrEP delivery model for FSWs.**

**REVIEWER 2**

Thank you for the opportunity to review this manuscript; it is an exciting exploration of client-centered prevention for a highly exposed population. Beyond the high importance and innovation of this work, a key strength is the substantial preliminary research around attributes and levels to inform the DCE. I raise below points of confusion or weakness for further consideration.

**Response**: *We sincerely appreciate the reviewer’s positive feedback and recognition of the importance and innovation of this work. Thank you for highlighting the strengths of our study, particularly the substantial preliminary research that informed the DCE. We also appreciate your thoughtful suggestions and have carefully considered the points raised for further refinement of the manuscript.*

1.Target population and validity of findings. There is a discordance between the stated aims / interpretation of the findings and the sample collected. The sample collected includes PrEP-experienced FSW provided care at a specialty clinic, but the paper as framed as applicable to FSW in Uganda more broadly. The difference is highly relevant in considering people's preferences for health services - the actual sample is highly selected based on these preferences, and the experience at the clinic likely in turn informs future preferences. While the introduction does not provide specific numbers for proportion of FSW in Uganda trying PrEP and staying on PrEP, it does state that PrEP use has been fairly low and persistence quite low. This suggests that the sample analyzed, which includes many long-term PrEP users, is likely to differ from the overall population of FSW in key ways related to health service use. The reliance on MARPI similarly will distinguish the sample from a broader target population in that experiences there are quite positive (per the Discussion section) in ways that 'preference for a clinic and HCW delivery' could obfuscate - preferences for clinics and HCW could be quite different for those picturing a typical public clinic. I would strongly suggest reframing the study objective and the interpretation of the finding to reflect a primary interest in supporting PrEP persistence, which fits with the choice of limiting the sample to PrEP-experienced FSW. The potential generalizability of these findings to FSW who have not already overcome barriers to PrEP and/or who don’t have access to MARPI should be a major focus of the discussion section.

**Response**: *Thank you for this thoughtful comment. We acknowledge the potential limitations in the generalizability of our findings due to the sample composition and the role of MARPI in providing PrEP services. MARPI has been a key player in PrEP implementation since 2017, offering both facility-based and community-based models of care, which have expanded access to FSW across Uganda. While MARPI’s services may attract individuals who are more engaged with healthcare, its national reach and integration into Uganda’s public health system make it a relevant setting for understanding PrEP preferences and persistence among FSW. In response to this valuable feedback, we have reframed the study objective and interpretation of findings to better reflect our focus on supporting PrEP persistence among PrEP-experienced FSW. Additionally, we have expanded the discussion to acknowledge potential differences in preferences and access barriers for FSW who have not yet initiated or remained on PrEP and those outside MARPI-supported programs. These revisions provide a clearer distinction between our sample and the broader FSW population while ensuring that the findings remain applicable to efforts aimed at improving PrEP uptake and retention nationwide.*

**To address these gaps, our study sought to identify Ugandan FSW preferences attending the MARPI clinic for a PrEP delivery model that offered structured options for location, services provided, and adherence support *(pages 3-4, lines 92 - 95).***

**In as much as most FSWs preferred healthy facility-based PrEP delivery, some preferred community-based healthcare services that involve community providers and peers rather than solely facility-based options, as this can help overcome stigma and discrimination barriers[1]. This divergence in preference contradicts the DCE homogenous assumption across the sample that preferences among individuals can vary greatly by previous experience. The plausibility of this assumption is limited to diverse populations *(pages 15, lines 328 - 331).***

2. DCE design: the selected attributes and levels may not directly violate the assumption of independence, but they certainly give rise to some convoluted options, like receiving PrEP from a peer at a health facility, or a health care worker giving PrEP to a peer to provider to the respondent. The presentation in the attached survey file seems to exacerbate this confusion, labeling the choices based on one attribute and leaving issues like how the PrEP picked up by a peer from a pharmacist actually gets to the respondent unanswered. (Other issues like referring to pharmacist in some settings and HCW in others, while these are the same level in the underlying design, are also concerning.) The ultimate design and certainly the analytic approach seem to move this experiment away from a traditional DCE, where each attribute is independent and assessed as its own contributor towards preference, and towards a conjoint analysis of overall models rather than separate attributes. Please provide further description and justification of this approach, including evidence from the preliminary assessments that respondents were able to assess each of the 4 attribute-levels within the choice sets as presented. Alternatively, please provide the theoretical basis or other reference material for this as a distinct type of preference experiment, with choice task presentation and analytic methods defined accordingly.

**Response**: *We thank the reviewer for this insightful feedback. We appreciate the opportunity to clarify and improve upon the design of our discrete choice experiment (DCE). We understand the concern regarding the potential confusion stemming from the selected attributes and levels, particularly in relation to the delivery models, such as receiving PrEP from a peer at a health facility or from a healthcare worker who administers PrEP to a peer. These choices do introduce complexities in understanding the roles and interactions between the respondent, healthcare workers, peers, and pharmacists. We endeavored to ensure that the pictorial presentations distinguished between the pharmacist and healthcare worker, given that both fall under the attribute of PrEP provider. To address this, we revised the presentation of the choice sets to ensure clarity regarding the flow of services (e.g., how a peer receives PrEP from a pharmacist and delivers it to the respondent). We also ensured consistent terminology throughout the survey to prevent confusion and make the roles of pharmacists and healthcare workers more distinct. Additionally, we provided clearer descriptions and explanations of the process within the survey itself so respondents could better assess each attribute and its levels.*

*Regarding the comment about the potential shift towards conjoint analysis, we agree that this aspect of the design warrants further clarification. While traditional DCEs generally treat attributes as independent and evaluate them separately, our design intended to account for the interaction between different service delivery models. However, we acknowledge that this approach could be perceived as moving toward a conjoint analysis model, where the overall preference for a service delivery model is assessed rather than the individual attributes. To clarify this, we provided a detailed explanation of our rationale for selecting this approach, highlighting the importance of understanding how the interaction between different service delivery models influences the respondents' preferences, as below (page 7, lines 156-161).*

**Generation of choice sets**

**We utilized a fractional factorial (D-efficient) design to generate choice sets that were optimally balanced within the given constraints. This method was chosen due to the large number of variable attributes, each with varying levels. Additionally, we did pilot testing to reduce the number of hypothetical delivery models presented to participants and avoid respondent fatigue.**

*We hope to include evidence from preliminary assessments in our future paper showing that respondents could comprehend and evaluate each attribute-level combination as presented in the choice sets while assessing the feasibility and effectiveness of the preferred model.*

3. Analysis: as mentioned in the prior point, the analysis presented diverges from traditional DCE analysis, which estimates utilities for each attribute (for the full sample and/or classes therein) in order to quantify the relative importance of the attributes and the relative preference for each level. It is then possible to estimate uptake of specific models combining attribute levels. The current approach jumps directly to assessment of pre-defined combination of attribute levels in a way that is not fully described, such that I am unable to assess its rigor and interpretability. What was the full set of models tested - every permutation of the 4 attributes / 13 levels? And then a subset of them was selected to be shown here based on some sort of ‘backwards selection’? How are readers to interpret these utility values? There is not enough detail to understand what was down and how to interpret it. It is striking that the authors mention in the limitations section that the opt out was the single most selected option, but these results are not presented in the Results section itself and would be impossible to read from the Tables shown, where the opt out option is fixed at 0.50 as the reference value. The analysis I would expect to see would describe the counts of respondents (including quality checks, see below) and then provide an overall or by-class utility model to provide the relative utility of each level and the heterogeneity of these preferences. The importance of each attribute would be quantified so that we can better understand even in context of these overall models’ weather location vs. reminder vs. person is primarily driving choices. Then specific models like those used here could be tested to estimate relative preference or expected uptake. It’s possible the process presented here did address some of these steps, but it’s not possible for me to really understand it at present, and overall, it does not seem to match the design process of creating a DCE with distinct attributes and levels.

**Response**: *We appreciate this thoughtful and detailed feedback. We greatly appreciate your observations and understand the concerns about the analysis approach, particularly regarding its divergence from traditional DCE methods. We agree that the traditional DCE methodology, which estimates utilities for each attribute (either for the full sample or subgroups) and quantifies the relative importance of each attribute and level, is an important aspect of understanding respondents’ preferences. As mentioned above, this approach provides the necessary foundation for interpreting how different levels of each attribute contribute to overall preferences and predicting the uptake of specific service delivery models. In the current analysis, we utilized a fractional factorial (D-efficient) design to generate choice sets that were optimally balanced within the given constraints^[2]^. Specifically, the pre-defined combinations were selected based on our prior research, which included a systematic review and qualitative studies, ensuring that the analysis aligned with the research objectives and addressed the key variables of interest. We also clarified every permutation of the 4 attributes and 13 levels that were tested (page 4, lines 118 -125).*

**Attributes and levels**

**We conducted a systematic review and qualitative study to identify DCE attributes^[3, 4]^.** **These findings were used to inform our study's final selection of attributes (Table 1). To create the choice sets, we employed a fractional factorial design; fractional D-deficiency designs are valuable for optimizing experimental efficiency when full factorial designs are not feasible. We relied on assumptions about factorial structure, D-efficiency, balance, confounding, additivity, variance homogeneity, and sample size, which resulted in eight sets**

*Regarding the utility values, we used the mlogit command to estimate the multinomial logit model and calculate the utility scores for each alternative in STATA 17.* *Utility scores are numerical values that reflect how much "value," "preference," or "satisfaction" a person assigns to a particular outcome or choice for our case, the PrEP delivery model. Utility scores (usually range between* ***0 and 1****. The higher the utility score, the greater the preference) Pages 9, lines 217-221. We ensured that the presentation of results, included utility values, reflects the full set of models tested and provides the necessary context for understanding their meaning see Table 4 page 11, line 271.*

**Table 4: Utility scores of choosing PrEP delivery**

| **#** | **Model** | **Median utility** | **IQR** |
| --- | --- | --- | --- |
| 1 | **Health facility/HCW/in-person/SMS** | **0.867** | **0.823, 0.941** |
| 2 | Home/peer/in-person/phone call | 0.749 | 0.646, 0.787 |
| 3 | Home/HCW/CHW/phone call | 0.727 | 0.605, 0.800 |
| 4 | Hotspot/HCW/CHW/phone call | 0.678 | 0.490, 0.715 |
| 5 | Home/HCW/in-person/SMS | 0.674 | 0.617, 0.748 |
| 6 | Health facility/peer/in-person/phone call | 0.579 | 0.470, 0.661 |
| 7 | Home/peer/in-person/None | 0.568 | 0.311, 0.674 |
| 8 | Health facility/HCW/peer/SMS | 0.558 | 0.471, 0.608 |
| 9 | Community/pharmacist/peer/SMS | 0.547 | 0.320, 0.591 |
| 10 | Health facility/HCW/peer/phone call | 0.504 | 0.431, 0.538 |
| 11 | Opt-out (None of the models) | 0.500 | 0.500, 0.500 |
| 12 | Hotspot/peer/in-person/SMS | 0.478 | 0.246, 0.561 |
| 13 | Community/HCW/CHW/None | 0.470 | 0.280, 0.526 |
| 14 | Hotspot/HCW/in-person/phone call | 0.460 | 0.237, 0.564 |
| 15 | Community/peer/in-person/ phone call | 0.456 | 0.438, 0.610 |
| 16 | Hotspot/HCW/in-person/SMS | 0.448 | 0.383, 0.629 |

*In addition, we acknowledge that the opt out model was not the most frequently selected model, based on the utility scores, that this statement was included in error. The statement been omitted. page 16, lines 368 – 369.*

4.The order of the methods section is a little jumbled - the information on the selection of attributes and levels is presented twice, as is the generation of choice sets, with information on data collection sandwiched between, and with more details on second presentation. I recommend streamlining for greater clarity.

**Response**: *We would like to clarify the distinction between the two tables presented. Table 1 outlines the attributes selected based on the systematic review and the qualitative studies conducted earlier in our research. These attributes served as the foundation for the subsequent analysis. In contrast, Table 2 presents the choice sets, which were developed using STATA software. These choice sets were constructed to facilitate the modeling and analysis processes discussed in our present study* *(see page 6, lines 130-134).*

**Table 1: DCE attributes and levels based on a systematic review and qualitative findings and expert panel review**

| **No.** | **Attribute** | **Level** | **Attribute definition for this study** |
| --- | --- | --- | --- |
| **1.** | **PrEP provider** | 1. Health worker | This can be a doctor, nurse, PrEP counselor, or other healthcare provider |
|  |  | 2. Peer | An FSW who is on PrEP and serves as a role model to the other FSW |
|  |  | 3. None | No preference for either |
| **2.** | **Place of PrEP delivery** | 1. Home | A place where the FSW lives |
|  |  | 2. Community | A designated place in the community where health workers come and offer HIV testing services and drug refills |
|  |  | 3. Health facility | Health care setting that provides PrEP |
|  |  | 4. Hotspot | A specific location where FSWs gather and transact sex |
| **3.** | **Delivery channel** | 1. In-person visits | FSW picks their own PrEP drugs |
|  |  | 2. Family member | A brother, spouse, sister, or member of the extended family picks the drug |
|  |  | 3. Peer | An FSW who is on PrEP and serves as an example to the other FSW |
| **4.** | **Additional support** | 1. Phone call reminder | A text message sent to a mobile phone 30 minutes before taking PrEP |
|  |  | 2. SMS reminder | A telephone call made to a mobile phone 30 minutes before taking PrEP |
|  |  | 3. None | No preference for either |

*SMS (Short message service)*

**Table 2: DCE choice sets with neither option**

| Choice set | Alternative | Place of PrEP Delivery | PrEP Provider | Delivery Channel | Additional support |
| --- | --- | --- | --- | --- | --- |
| 1 | I | Hotspot | None | Peer | SMS reminder |
|  | II | Home | Peer | In-person | call reminder |
|  | III | Community | Peer | Family member | SMS reminder |
|  | IV | Neither |  |  |  |
| 2 | I | Home | HW | Family member | call reminder |
|  | II | Community | Peer | Family member | SMS reminder |
|  | III | Health Facility | None | In-person | SMS reminder |
|  | IV | Neither |  |  |  |
| 3 | I | Hotspot | None | Family member | call reminder |
|  | II | Health Facility | HW | Peer | SMS reminder |
|  | III | Community | Peer | Family member | SMS reminder |
|  | IV | Neither |  |  |  |
| 4 | I | Home | HW | In-person | SMS reminder |
|  | II | Community | None | Peer | call reminder |
|  | III | Community | Peer | Family member | SMS reminder |
|  | IV | Neither |  |  |  |
| 5 | I | Health Facility | HW | Peer | call reminder |
|  | II | Community | Peer | Family member | SMS reminder |
|  | III | Hotspot | Peer | In-person | SMS reminder |
|  | IV | Neither |  |  |  |
| 6 | I | Health Facility | Peer | In-person | call reminder |
|  | II | Hotspot | HW | Family member | None |
|  | III | Community | Peer | Family member | SMS reminder |
|  | IV | Neither |  |  |  |
| 7 | I | Community | Peer | Family member | SMS reminder |
|  | II | Home | Peer | Peer | None |
|  | III | Hotspot | HW | Family member | call reminder |
|  | IV | Neither |  |  |  |
| 8 | I | Community | Peer | Family member | SMS reminder |
|  | II | Hotspot | HW | In-person | SMS reminder |
|  | III | Home | Peer | Peer | call reminder |
|  | IV | Neither |  |  |  |

*The D-efficiency of the design was 88.4, % which is above the 80% recommended score*

5. What was the mode of data collection, paper prior to the entry into Excel? Or collected on tablet / laptop directly into Excel? Please specify this and note any data quality checks, given that these are not standard survey collection tools with built in quality checks

**Response**: *The data collection section has been updated in the manuscript (see pages 7-8, lines 177-194).*

**Data collection**

**The data for this study were initially collected through a paper-based (questionnaire) survey, which was later transcribed into Microsoft Excel for further analysis. FSW filled out the questionnaires manually, with the assistance of research assistants, and later, trained data entry personnel entered the responses into Excel. This approach was particularly useful as FSW had limited ability to use digital tools and preferred to use paper-based tools.**

6. What DCE quality checks were built in, regarding timing, repeat choice, internal validity? What tests were performed for straight-line responses or other signs of invalid responses? If none, please note as a limitation.

**Response**: *The data quality section has been revised as follows (see page 9, lines 228-240).*

**Data quality control**

**We monitored the duration of participant responses to ensure that the choices were being made thoughtfully. Responses completed significantly faster than average (within 5 minutes) were flagged for further review, as they indicated that the participant was not properly considering the options. To assess whether participants were paying attention, we included a set of identical choice sets within the questionnaire. This method helped identify if FSW were selecting the same option repeatedly without regard to the alternatives. FSW who consistently made the same choice in repeat sets were flagged for potential invalidity. For straight-line responses, where FSW selected the same answer across all choice sets, we used data analysis to identify patterns that indicate a lack of engagement or random selection. If FSW responses appeared to follow a straight line or exhibited no variability, we treated this as a potential sign of invalidity and excluded such responses from the analysis.**

7. Please consider the DCE assumptions in the discussion section and assess the evidence and plausibility of these assumptions.

**Response**: *The Discussion section has been amended as recommended to include two of the DCE assumptions and their plausibility. The assumptions considered include i) assumptions of homogenous preferences across the sample and ii) the assumption of accurate and complete information, and are highlighted below (page 14, lines 292-313).*

**DISCUSSION**

This discrete choice experiment, conducted with cisgender FSWs in Kampala, Uganda, utilized a cross-sectional survey to determine the most end-user-centric PrEP delivery model. FSW preferred delivery through a health provider at the sex worker-friendly MARPI clinic as the next best option, with additional support in the form of SMS reminders as the next best option for enhancing adherence and engagement with the PrEP regimen. These findings provide valuable insights into the preferences of FSWs for PrEP delivery, emphasizing the importance of provider involvement and tailored support in improving access and uptake within this key population. Findings from the qualitative phase revealed that healthcare providers at the clinic were perceived as friendly and non-judgmental, creating a comfortable environment for these women [4]. They were also seen as capable of maintaining confidentiality while delivering PrEP services. Participants reported feeling welcomed, included, and safe at the MARPI clinic, which encouraged them to continue utilizing its services ^[3, 4^].

**The assumption of accurate and complete information is similar to findings of prior studies** that FSWs appreciate PrEP introduction within familiar and trusted "friendly" clinics tailored for sex workers and value positive encouragement from clinic staff ^[5-7].^ The plausibility of this assumption is that, whereas FSW might not have had accurate and complete information on all the PrEP delivery models, our DCE mitigated this issue by focusing on key attributes, allowing FSW to make choices based on the most relevant factors. In our study, healthcare workers were perceived as knowledgeable individuals with specialized knowledge about PrEP. This preference for healthcare workers as PrEP providers is consistent with previous research conducted in Uganda, which found that PrEP delivery based at health facilities required healthcare providers to have sufficient knowledge and confidence in discussing antiretroviral medications for HIV prevention with clients [8]. Similar results have been demonstrated in family health programs in Brazil, Bangladesh, and Nepal, in which health workers positively influenced health by serving as entry points, bridges, and connectors to healthcare services, systems, and resources ^[9-11].^ Our results emphasize the importance of involving healthcare workers in PrEP delivery for FSWs ^[4]^.

In as much as most FSWs preferred healthy facility-based PrEP delivery, some preferred community-based healthcare services that involve community providers and peers rather than solely facility-based options, as this can help overcome stigma and discrimination barriers ^[1]^**. This divergence in preference contradicts the DCE homogenous assumption across the sample that preferences among individuals can vary greatly by previous experience. The plausibility of this assumption is limited to diverse populations.** The World Health Organization recommends differentiated approaches for delivering PrEP services, prioritizing the individual and community ^[12]^. These approaches are adapted to the specific needs and preferences of individuals who may benefit from PrEP. Implementing these differentiated services can improve PrEP acceptability and accessibility and support its ongoing use and effectiveness ^[13]^. Community-based delivery options such as pharmacies, community organizations, drop-in centers, and mobile clinics complement facility-based care by providing strong linkages and referral pathways for those seeking treatment ^[14]^. Our findings underscore the importance of customizing differentiated service delivery models for FSWs.

8. The first paragraph of the discussion refers more to the previous studies than this study. Please provide a succinct summary of the major insights of the current study before placing them in perspective later in the discussion section.

**Response**: *We thank the reviewer for this invaluable guidance. The first paragraph of the discussion section has been revised to include a summary of the study’s key findings (see page 14, lines 287-301).*

**DISCUSSION**

**This discrete choice experiment, conducted with cisgender FSWs in Kampala, Uganda, utilized a cross-sectional survey to determine the most end-user-centric PrEP delivery model. FSW preferred delivery through a health provider at the sex worker-friendly MARPI clinic as the next best option, with additional support in the form of SMS reminders as the next best option for enhancing adherence and engagement with the PrEP regimen. These findings provide valuable insights into the preferences of FSWs for PrEP delivery, emphasizing the importance of provider involvement and tailored support in improving access and uptake within this key population. Findings from the qualitative phase revealed that healthcare providers at the clinic were perceived as friendly and non-judgmental, creating a comfortable environment for these women ^[4]^. They were also seen as capable of maintaining confidentiality while delivering PrEP services. Participants reported feeling welcomed, included, and safe at the MARPI clinic, which encouraged them to continue utilizing its services ^[3, 4]^.**

9. The Discussion section mentions 150 as a standard DCE size without citation; DCE sample size is fundamentally dependent on the design, so reference to a standard sample size is not appropriate.

**Response**: As advised, w*e have revised the Discussion section to more accurately reflect how a sample size of 150–200 would adequately answer the research objective. We have also included a reference as requested. The revision reads as follows. (see page 16, lines 359-361)*

**Additionally, our results are based on a robust sample size of >200 FSWs, surpassing the recommended number of 150 for DCEs that has sufficient statistical power to detect significant effects. (Lancsar E, Louviere J. Conducting discrete choice experiments to inform healthcare decision making. Pharmacoeconomics. 2008;26(8):661-677).**

Minor
Note that the rows for Table 1. 4. Additional support are flipped (phone call vs. text)
It’s difficult to interpret ‘none’ within the attributes - it’s defined as no preference between them, but on its face would be read as a preference against both, so ‘no provider’, e.g. self service, and no reminder. I would revise Table 1 to indicate that it means (at least based on the attached tool) ‘no provider’ ‘no reminder’ rather than no preference.

**Response**: *We appreciate the helpful feedback and support in clarifying this point. The term "no preference" has been updated to "no reminder," as noted in the table below. (see page 5, lines 130-132)*

**Table 1: DCE attributes and levels based on a systematic review and qualitative findings and expert panel review**

| **No.** | **Attribute** | **Level** | **Attribute definition for this study** |
| --- | --- | --- | --- |
| **1.** | **PrEP provider** | 1. Health worker | This can be a doctor, nurse, PrEP counselor, or other healthcare provider |
|  |  | 2. Peer | An FSW who is on PrEP and serves as a role model to the other FSW |
|  |  | 3. None | No preference for either |
| **2.** | **Place of PrEP delivery** | 1. Home | A place where the FSW lives |
|  |  | 2. Community | A designated place in the community where health workers come and offer HIV testing services and drug refills |
|  |  | 3. Health facility | Health care setting that provides PrEP |
|  |  | 4. Hotspot | A specific location where FSWs gather and transact sex |
| 3. | **Delivery channel** | 1. In-person visits | FSW picks their own PrEP drugs |
|  |  | 2. Family member | A brother, spouse, sister, or member of the extended family picks the drug |
|  |  | 3. Peer | An FSW who is on PrEP and serves as an example to the other FSW |
| 4. | **Additional support** | 1. Phone call reminder | A text message sent to a mobile phone 30 minutes before taking PrEP |
|  |  | 2. SMS reminder | A telephone call made to a mobile phone 30 minutes before taking PrEP |
|  |  | 3. None | No reminder |

Ruth Mpirirwe

02.05. 2025

1.	Chautrakarn S, Rayanakorn A, Intawong K, Chariyalertsak C, Khemngern P, Stonington S, et al. Prep stigma among current and non-current prep users in Thailand: a comparison between hospital and key population-led health service settings. Frontiers in Public Health. 2022;10:1019553.

2.	Walker JL, Wang Y, Thorhauge M, Ben-Akiva M. D-efficient or deficient? A robustness analysis of stated choice experimental designs. Theory and Decision. 2018;84:215-38.

3.	Mpirirwe R, Segawa I, Ojiambo KO, Kamacooko O, Nangendo J, Semitala FC, et al. HIV pre-exposure prophylaxis uptake, retention and adherence among female sex workers in sub-Saharan Africa: a systematic review. BMJ open. 2024;14(4):e076545.

4.	Mpirirwe R, Mujugira A, Walusaga H, Ayebare F, Musanje K, Ndugga P, et al. Perspectives of female sex workers on HIV pre-exposure prophylaxis delivery in Uganda: A qualitative study. Research Square. 2024.

5.	Busza J, Chiyaka T, Musemburi S, Fearon E, Davey C, Chabata S, et al. Enhancing national prevention and treatment services for sex workers in Zimbabwe: a process evaluation of the SAPPH-IRe trial. Health policy and planning. 2019;34(5):337-45.

6.	Nakanwagi S, Matovu JK, Kintu BN, Kaharuza F, Wanyenze RK. Facilitators and barriers to linkage to hiv care among female sex workers receiving hiv testing services at a community-based organization in Periurban Uganda: A Qualitative Study. Journal of sexually transmitted diseases. 2016;2016.

7.	Nnko S, Kuringe E, Nyato D, Drake M, Casalini C, Shao A, et al. Determinants of access to HIV testing and counselling services among female sex workers in sub-Saharan Africa: a systematic review. BMC public health. 2019;19(1):1-12.

8.	Muwonge TR, Nsubuga R, Ware NC, Wyatt MA, Pisarski E, Kamusiime B, et al. Health care worker perspectives of HIV Pre-exposure prophylaxis service delivery in central Uganda. Frontiers in Public Health. 2022;10:658826.

9.	Chen L, Evans T, Anand S, Boufford JI, Brown H, Chowdhury M, et al. Human resources for health: overcoming the crisis. The lancet. 2004;364(9449):1984-90.

10.	Haines A, Sanders D, Lehmann U, Rowe AK, Lawn JE, Jan S, et al. Achieving child survival goals: potential contribution of community health workers. The lancet. 2007;369(9579):2121-31.

11.	Macinko J, Guanais FC, De Souza MDFM. Evaluation of the impact of the Family Health Program on infant mortality in Brazil, 1990–2002. Journal of Epidemiology & Community Health. 2006;60(1):13-9.

12.	Organization WH. Differentiated and simplified pre-exposure prophylaxis for HIV prevention: update to WHO implementation guidance: technical brief. 2022.

13.	Lancaster KE, Lungu T, Bula A, Shea JM, Shoben A, Hosseinipour MC, et al. Preferences for pre-exposure prophylaxis service delivery among female sex workers in Malawi: a discrete choice experiment. AIDS and Behavior. 2020;24:1294-303.

14.	Mugambi ML, Odhiambo BO, Dollah A, Marwa MM, Nyakina J, Kinuthia J, et al. Women's preferences for HIV prevention service delivery in pharmacies during pregnancy in Western Kenya: a discrete choice experiment. Journal of the International AIDS Society. 2024;27:e26301.
